# Duodenal Ulcer and Gastrointestinal Bleeding as a Late Complication of Over-The-Scope Clip Placement

**DOI:** 10.14309/crj.0000000000001657

**Published:** 2025-04-04

**Authors:** Aditya Avula, Ahmed Al-Chalabi, Erin Jenkins

**Affiliations:** 1 Department of Internal Medicine,Cr eighton University, Omaha, NE; 2 Department of Gastroenterology, Creighton University, Omaha, NE

## CASE REPORT

**Figure 1.**
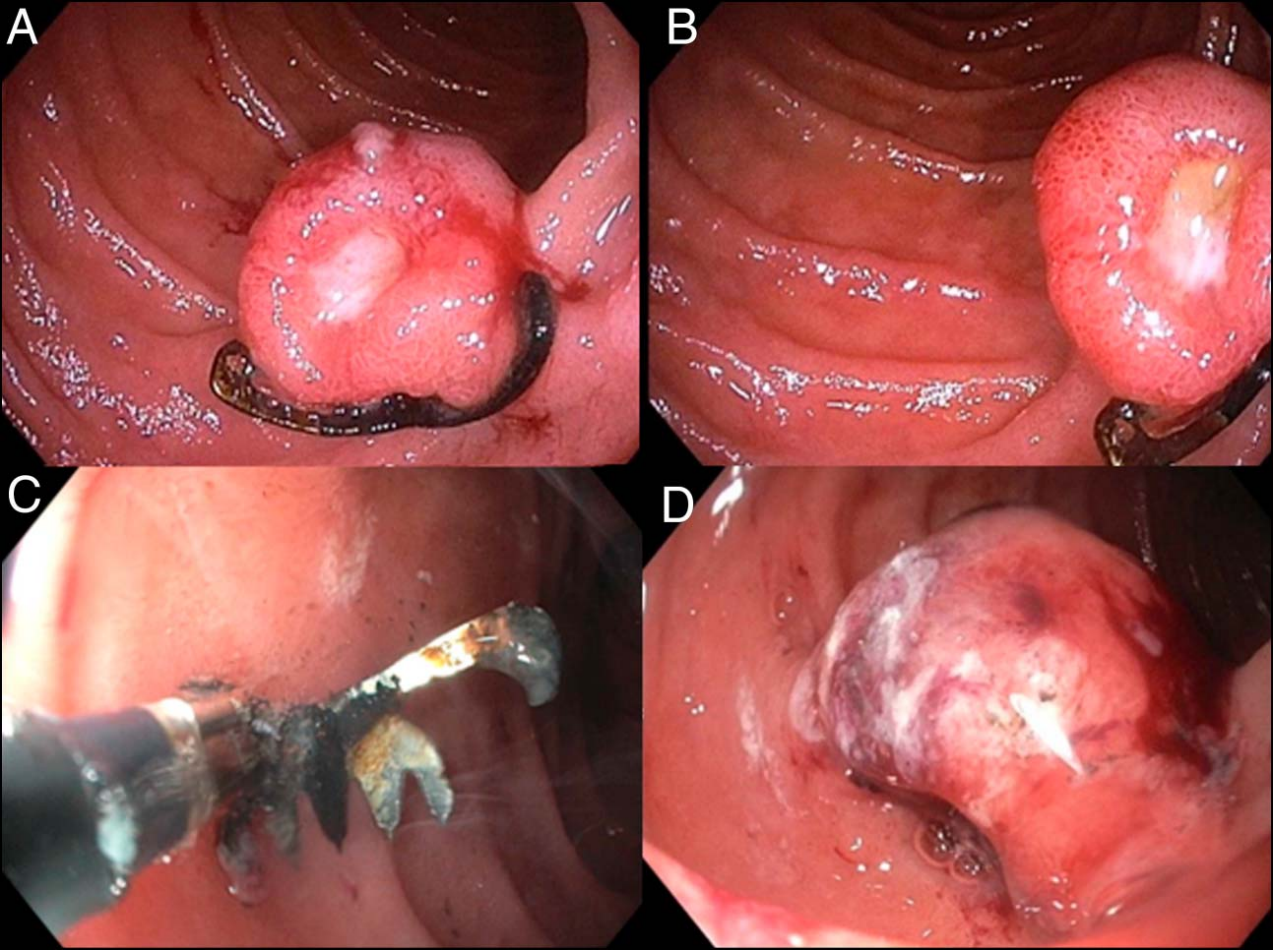
(A and B) Retained OTSC from prior endoscopy found in the second portion of the duodenum that was found to be surrounding an oozing ulceration. This ulceration was determined to be the source of the patient's melena. (C) Bipolar cutting instrument (remOVE direct current Cutter) used to fragment the retained OTSC into 3 pieces. (D) Duodenal ulceration post-OTSC removal with residual oozing. OTSC, over-the-scope clip.

These images (Figure 1) are from a 70-year old man who presented with a 5-day history of melena and concomitant lightheadedness. Thirty-one months earlier, he experienced similar symptoms and was found to have a duodenal Dieulafoy lesion. This lesion was managed through the application of an over-the-scope clip (OTSC) (Ovesco Endoscopy USA), achieving hemostasis and subsequent clinical improvement.

Endoscopy on this presentation revealed a 15 mm mucosal nodule with an oozing ulcer in the second portion of the duodenum. There was a foreign body at the base of the nodule consistent with a retained OTSC from previous endoscopy (Figure 1). Biopsies of the ulcer showed no evidence of malignancy. This ulceration was deemed to be the bleeding source, and repeat endoscopy was performed to remove the OTSC, which was fragmented into 3 pieces using a bipolar cutting instrument (remOVE direct current Cutter) and retrieved with grasping forceps (Figure 1). The patient's melena resolved shortly after this intervention.

While acute adverse events associated with OTSC implementation have been documented, prolonged complications have not been widely reported. This case illustrates the potential for chronic ulceration and subsequent hemorrhage in association with a retained OTSC, and the importance of familiarity with removal techniques.

## DISCLOSURES

Author contributions: A. Avula and E. Jenkins: design of the work; acquisition, analysis, and interpretation of data for the work. A. Avula: drafting and reviewing intellectual content including formations of text and images; submission of final text and imagery. E. Jenkins: design of the work; acquisition of endoscopic images and initial endoscopy. A. Al-Chalabi: design of the work; initial endoscopy, improving text and images; draft revision, and review of final text and imagery. A. Avula is the article guarantor.

Financial disclosure: None to report.

Previous presentation: This case was presented at the American College of Gastroenterology (ACG) Annual Scientific Meeting and Postgraduate Course; October 27, 2024; Philadelphia, PA.

Informed consent was obtained for this case report.

